# Opportunistic Ultrasound Screening for Abdominal Aortic Aneurysm

**DOI:** 10.3400/avd.oa.23-00110

**Published:** 2024-04-10

**Authors:** Yasuharu Funamizu, Hitoshi Goto, Ayaka Oda, Takashi Miki, Yoshifumi Saijo

**Affiliations:** 1 Clinical Physiology Center, Tohoku University Hospital, Sendai, Miyagi, Japan; 2 Department of Vascular Surgery, South Miyagi Medical Center, Ogawara, Miyagi, Japan; 3 Department of Clinical Practice and Support, Hiroshima University Hospital, Hiroshima, Hiroshima, Japan; 4 Graduate School of Biomedical Engineering, Tohoku University, Sendai, Miyagi, Japan

**Keywords:** screening, abdominal aortic aneurysm, ultrasound

## Abstract

**Objective**: In patients with abdominal aortic aneurysm (AAA), early detection and optimal elective treatment before rupture are desirable. In the absence of an established public screening system, opportunistic screening during ultrasound examination for another purpose might be efficacious. The aim of this study was to evaluate the efficacy of opportunistic screening for AAA.

**Methods**: This prospective multicenter observational study enrolled patients who were scheduled to undergo ultrasound for reasons other than AAA. After the ultrasound for the original purpose, evaluation of the abdominal aorta was added. If the abdominal aorta was clear enough for measurement, its diameter and shape were recorded. Furthermore, information on comorbidities was collected for each patient.

**Results**: A total of 10325 patients (echocardiography: 6150; abdominal ultrasound: 4162) from 16 institutions were enrolled. The abdominal aorta was well visualized in 92.9% of patients who underwent echocardiography. Among 9791 patients, AAA was diagnosed in 122 (1.3%) (107 fusiform and 15 saccular), with a diameter range of 30–63 mm. The diagnostic rate increased with age. On multivariate analysis, older age, male sex, coronary artery disease, peripheral arterial disease, and smoking habituation were the risk factors for AAA.

**Conclusion**: Opportunistic screening for AAA was efficacious.

## Introduction

Ruptured abdominal aortic aneurysm (RAAA) is a fatal condition and a leading cause of death in Western countries.^1)^ In Japan, RAAA is also thought to be an important issue, although the precise statistics had been unknown. RAAA was reported to have a high mortality rate even after surgery was performed, but elective surgery had good surgical outcome.^2)^ Therefore, detection and proper treatment of AAAs before rupture would be the most effective way to prevent this catastrophic state. However, because most AAAs remain asymptomatic, the chances of diagnosis are low, unless patients undergo screening.

Screening programs have been demonstrated to reduce the frequency of rupture and AAA-related deaths by providing early diagnosis and allowing elective surgical treatment. A meta-analysis of four population-based randomized controlled trials showed that AAA screening of men ≥65 years old significantly reduced AAA-related mortality.^3)^ Based on this evidence, several screening programs had been carried out in several countries.^4^^–^^6)^

In Japan, there is no public screening system, and most AAAs are detected incidentally during examinations for other purposes. Therefore, some cases of rupture were not previously diagnosed as AAA. Establishment of a new screening system requires time and labor; therefore, we thought that opportunistic screening during imaging for other indications might be easier and efficacious. The aims of this study were to evaluate the efficacy of opportunistic screening for AAA and to evaluate the prevalence and risk factors for AAA in our population.

## Materials and Methods

### Study design

This study was approved by the Institutional Review Board of Tohoku University, Sendai, Japan (No. 2015-1-685). This was a prospective multicenter observational investigation from April 2016 to December 2017 and was participated in by the following 16 institutions: Tohoku University Hospital, Aomori Prefectural Central Hospital, Mutsu General Hospital, Tsugaru Hoken Medical CO-OP Kensei Hospital, Omagari Kousei Medical Center, Akita Kousei Medical Center, Research Institute for Brain and Blood Vessels, Sanai Hospital, JR Sendai Hospital, Osaki Citizen Hospital, Kesennuma City Hospital, Southern Tohoku General Hospital (Iwanuma), Yamagata University Hospital, Yamagata Prefectural Shinjo Hospital, Nagai Clinic, and Southern Tohoku General Hospital (Koriyama).

Patients who were scheduled to undergo ultrasound for reasons other than AAA and those who consented verbally were enrolled. The indications for ultrasound included validation of cardiac function, screening of the hepatobiliary organs, and evaluation of hepatic disease. After the ultrasound was performed for the original purpose, additional evaluation of the abdominal aorta was performed. First, the abdominal aorta was judged if it could be well visualized for validation. If the detected abdominal aorta was clear enough to be measured, its diameter and shape were recorded. Most of the examinations were done by well-experienced sonographers. The medical history, including hypertension, diabetes mellitus (DM), coronary artery disease (CAD), cerebrovascular disease (CVD), peripheral arterial disease (PAD), chronic kidney disease (CKD), and smoking habituation, were asked from the patients during examination or obtained from the medical records. The exclusion criteria were the age <20 years; known AAA; solitary iliac artery aneurysm; AAA-related connective tissue disease, such as Marfan’s syndrome and Ehlers–Danlos syndrome; and patients who did not consent to participate in the study.

### Definitions

AAA was defined as an abdominal aorta with a fusiform shape and a diameter of ≥30 mm; an abdominal aorta with a saccular shape was defined as AAA, regardless of its diameter. Unilateral protruding of the aorta was defined as a saccular aneurysm, and the long axis including the normal aorta was defined as the aneurysm diameter. There were no strict criteria for the diagnosis of the comorbidities, because, in some patients, this information was obtained by history taking or from medical records.

### Statistical analysis

Categorical variables were presented as number (%) and were compared between groups using the chi-square test. Ordinal variables were presented as median ± standard deviation and were compared between groups using the Mann–Whitney U test. The optimal cutoff value for age was obtained using the Youden index based on the ROC analysis. The baseline variables that showed a univariate relationship with the detection of AAA were entered into the multivariate analysis. Multivariate logistic regression analysis was performed to identify the independent factors associated with the presence of AAA. All statistical analyses were performed by JMP Pro (SAS Institute Inc., Cary, NC, USA). The differences were considered significant at P <0.05.

## Results

### Study population and detection rate

During the study period, a total of 10325 patients (5730 men and 4595 women) underwent examination by echocardiography (n = 6150), abdominal ultrasound (n = 4162), or an unknown ultrasound category (n = 13). The abdominal aorta could be visualized well by echocardiography in 5712 (92.9%) patients and by abdominal ultrasound in 4078 (97.9%) patients, with a superior visualization rate by the latter (P <0.001). The detection rate was not different between men and women (94.8% versus 94.9%, respectively, P = 0.88).

After excluding cases with poorly visualized abdominal aorta (n = 534), the remaining 9791 patients were analyzed. The characteristics of the study population are shown in **Table 1**. The median age was 67 years (range, 20–101 years); there were 5432 men and 4359 women.

**Table:** Table 1 Characteristics of the patients

Generation	Number	Male/Female	HT (%)	DM (%)	PAD (%)	CKD (%)	CAD (%)	CVD (%)	Smoking (%)
20s	170	79/91	9 (5.3)	3 (1.8)	0 (0)	2 (1.2)	1 (0.6)	1 (0.6)	28 (16.5)
30s	428	261/167	59 (13.8)	22 (5.1)	4 (0.9)	11 (2.6)	8 (1.9)	4 (0.9)	141 (32.9)
40s	939	560/379	202 (21.5)	82 (8.7)	14 (1.5)	59 (6.3)	55 (5.9)	25 (2.7)	357 (38)
50s	1536	923/613	562 (36.6)	213 (13.9)	50 (3.3)	106 (6.9)	112 (7.3)	69 (4.5)	644 (41.9)
60s	2516	1475/1041	1207 (48)	500 (19.9)	114 (4.5)	202 (8)	317 (12.6)	187 (7.4)	939 (37.3)
70s	2483	1385/1098	1398 (56.3)	538 (21.7)	109 (4.4)	223 (9)	425 (17.1)	288 (11.6)	799 (32.2)
80s	1562	682/880	936 (59.9)	312 (20)	87 (5.6)	138 (8.8)	279 (17.9)	207 (13.3)	369 (23.6)
90 and over	157	67/90	96 (61.1)	23 (14.6)	12 (7.6)	15 (9.6)	21 (13.4)	31 (19.7)	39 (24.8)
Total	9791	5432/4359	4469 (45.6)	1693 (17.3)	390 (4)	756 (7.7)	1218 (12.4)	812 (8.3)	3316 (33.9)

HT: hypertension; DM: diabetes mellitus; PAD: peripheral arterial disease; CKD: chronic kidney disease; CAD: coronary artery disease; CVD: cerebrovascular disease

### Prevalence of AAA

**Figure 1:**
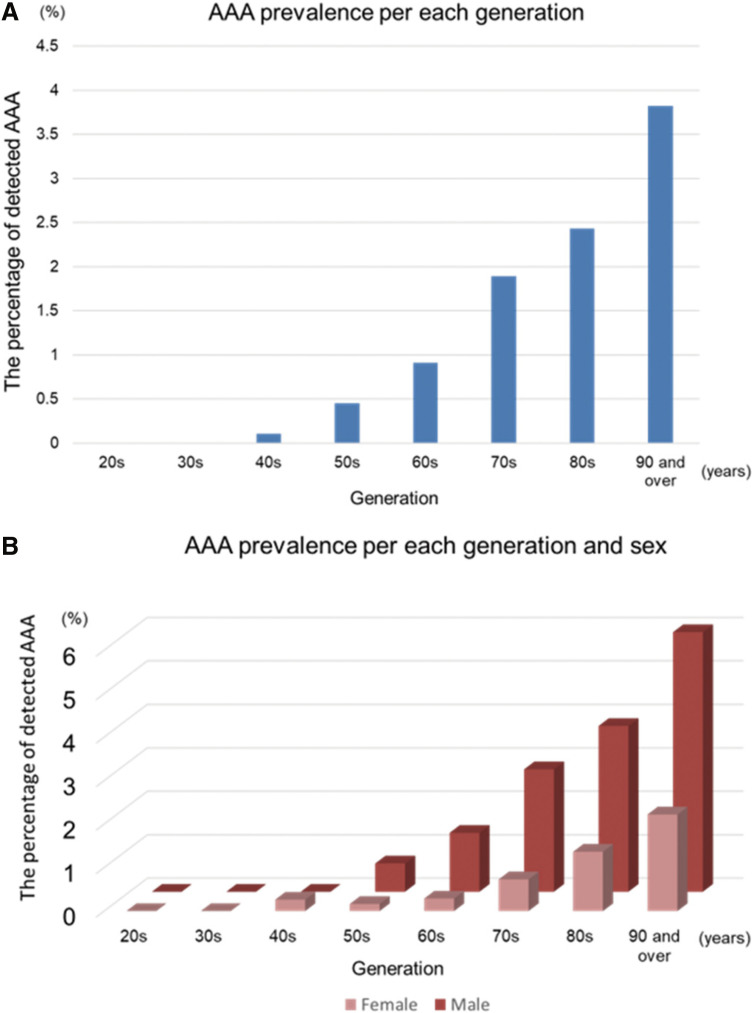
Fig. 1 AAA prevalence (**A**) per each generation and (**B**) per each generation and sex. AAA: abdominal aortic aneurysm

AAA was diagnosed in 122 (1.3%) of the 9791 patients. The diagnostic rate increased with age (**Fig. 1A**). To identify the optimal cutoff value for age at which AAA can be diagnosed effectively, ROC curve analysis was performed to calculate the sensitivity and specificity for predicting the prevalence by age. The optimal cutoff age for the detection of AAA was 73 years. The diagnostic rate of AAA was 2.4% for those aged ≥73 years (n = 3475). In addition, the diagnostic rate was significantly higher in men than in women (1.75% versus 0.64%, P <0.0001). Focus on men aged ≥65 years based on the screening criteria in the United States, the diagnostic rate was 2.7%. Furthermore, the diagnosis rate was 3.4% when narrowing down to smokers among men aged 65 years and older. The distribution of AAA detection by sex and age is shown in **Fig. 1B**.

### Diameter of AAA

**Figure 2:**
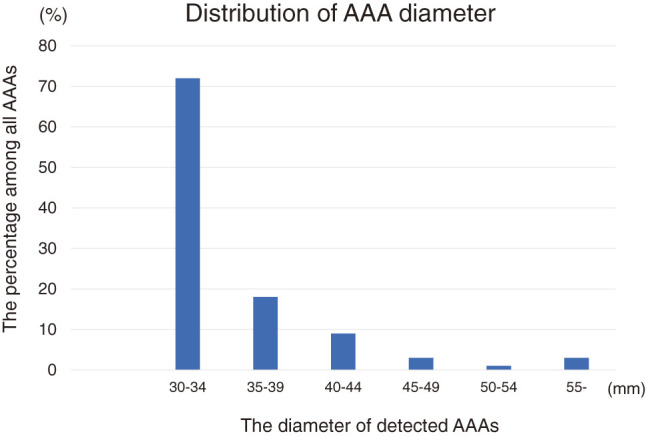
Fig. 2 Distribution of AAA diameter. The data refer to only fusiform aneurysms and exclude saccular aneurysms. AAA: abdominal aortic aneurysm

Among the AAAs detected in this study, 107 were fusiform and 15 were saccular. The diameter of the fusiform AAAs (range, 30–63 mm) was 30–39 mm in 90 cases, 40–49 mm in 12 cases, and >50 mm in four cases (**Fig. 2**). The long axis diameter of the saccular AAAs ranged from 31 to 60 mm. Although this study did not focus on iliac artery aneurysms, 18 patients were found to have iliac artery aneurysms, with a median diameter of 27 (range, 21–42) mm, of which four were with AAA and 14 without AAA.

### Comorbidities and risk factors

**Table 1** shows the comorbidities and smoking habituation of the study population. About half of the patients had hypertension, and one-third were smokers. **Table 2** shows the comparison of the comorbidities between the AAA detected and AAA undetected groups. Univariate analysis showed that age, sex, PAD, CAD, and smoking habituation were strongly related with AAA detection. In multivariate analysis excluding age and sex, CAD, PAD, and smoking habits showed high odds ratios for the detection of AAA. When divided by sex, high odds ratios were observed for CAD and PAD in men, whereas only CAD was strongly associated with AAA in women (data not shown).

**Table:** Table 2 Univariate and multivariate analyses of the risk factors associated with detected AAA

	Univariate analysis			Multivariate analysis		
	AAA (+) N = 122	AAA (−) N = 9791	P value			
				Odds ratio	95% CI	P value
Sex (male%)	77.9	55.2	<0.0001	2.35	1.47–3.77	0.0004
Age (y.o. median)	75	65	<0.0001※1	–	–	
Age (73 y.o. and over)	83	3392	<0.0001	3.98	2.69–5.90	<0.0001
HT	71	4398	0.0051			
DM	21	1672	0.98			
PAD	16	374	<0.0001	2.5	1.44–4.37	0.0148
CKD	19	737	0.001			
CAD	41	1177	<0.0001	2.28	1.52–3.41	<0.0001
CVD	15	797	0.11			
Smoking	66	3250	<0.0001	1.65	1.10–2.47	0.0155

※1 : Mann–Whitney U test.

AAA: abdominal aortic aneurysm; CI: confidence interval; y.o.: year old; HT: hypertension; DM: diabetes mellitus; PAD: peripheral arterial disease; CKD: chronic kidney disease; CAD: coronary artery disease; CVD: cerebrovascular disease

## Discussion

In this study, we showed the efficacy of opportunistic screening for AAA during echocardiography and abdominal ultrasound for other indications. In addition, we presented the AAA prevalence according to age and associated comorbidities. The efficacy of AAA screening has been reported by several studies.^1^^,^^3^^,^^7^^,^^8)^ Ali et al. reported that population-based ultrasound screening for AAA in asymptomatic men aged ≥65 years significantly reduced the rates of unexpected AAA-related mortality and rupture.^7)^ Similarly, the Canadian Task Force on Preventive Health Care analyzed four population-based RCTs and provided evidence that screening men aged 65–80 years had a sufficiently positive impact on reducing mortality, rupture, and emergency procedures, thereby, outweighing the risk of unnecessary elective procedures.^8)^ On the other hand, a multicenter randomized trial on 67770 men aged 65–74 years showed 42% reduction of AAA-related deaths in the ultrasound screening group, compared with the rate in the observation group.^9)^

In Japan, Ishikawa et al. found AAA in 0.3% of 10057 residents aged ≥60 years by ultrasound screening and concluded that screening, including the expected elective surgery, had cost benefits, compared with the estimated cost of no screening, including the expected emergent surgery.^10)^ One report mentioned that many patients with ruptured AAAs were not previously screened.^11)^ Indeed, many cases of ruptured AAA were unlikely to have been previously diagnosed, because public screening systems in our country are few. Therefore, it is important to establish a screening system.

Ultrasound had been reported to be useful for the diagnosis of AAA because of its high sensitivity and specificity.^9)^ Usually, ultrasound of the abdomen, including the abdominal aorta, is done using a convex probe, which has a wide field of visualization. On the other hand, clear detection of the abdominal aorta by the sector probe used in echocardiography might be controversial. Aboyans et al. reported that among 1407 patients who were screened for AAA by 108 cardiologists, the imaging quality of echocardiography was evaluated as excellent in 51.5%, good in 37.9%, fair in 7.3%, and poor in only 3.3%.^12)^ Another report on AAA screening by echocardiography mentioned that measurement of the abdominal aorta was feasible in 71% of 1300 patients with CAD but unsuitable for the remaining 29% because of obesity, body habitus, or the surgical wound after CABG.^13)^ In our study, although the detection rate was lower with the sector probe than with the convex probe, the abdominal aorta could be evaluated by echocardiography in 92.9%. The AAA detection rate may depend on the kind of ultrasound technology, sonographer technique, and patient condition, such as obesity or postoperative status. Nevertheless, AAA screening during echocardiography may be feasible. In fact, a meta-analysis reported a higher AAA prevalence among patients who underwent screening transthoracic echocardiography (TTE) than in the general population of contemporary epidemiological studies and mentioned that TTE could be a valuable tool for opportunistic AAA screening.^13)^

In the United States and Australia, the prevalence of AAA was reported to be as high as 8% in men and 2% in women aged ≥65 years.^14^^,^^15)^ A systematic review on Asians reported an AAA prevalence of 1.3%.^16)^ In Japan, there had been only few studies on AAA prevalence, which was reported to be 0.3% among 1591 farming community residents who were screened by ultrasound in the study by Adachi et al.^17)^ and 4.1% among 1731 hypertensive patients >60 years old who were screened by handy pocket echo in the study by Fukuda et al.^18)^ The difference in the reported AAA prevalence might be accounted for by differences in the characteristics and district of the target populations. In this study, although the characteristics of our cohort likely differed from those of the general population, we found that the prevalence of AAA was relatively high in men and increased with age in both men and women.

To increase the efficacy of screening, identification of risk factors and cases with high pretest probability is essential. Aging, male sex, and smoking are well-known risk factors of AAA and had been adopted as the screening criteria by many programs. In this study, diagnostic efficiency also increased when 65 years of age, male gender, and smoking were used as screening conditions.

On the other hand, on multivariate analysis excluding age and sex, only smoking habituation, CAD, and PAD were the independent predictors of AAA detection. Several studies have reported smoking as a risk factor for AAA expansion.^4^^,^^19)^ CAD was reported to be a risk factor for AAA by many studies^12^^,^^13^^,^^20^^,^^21)^ but was found to be not associated with AAA in a meta-analysis^22)^; therefore, there is room for further discussion on CAD. Similarly, PAD as a risk factor for AAA remains a topic of debate, and future accumulation of data is desired.^13^^,^^19^^,^^23)^ Interestingly, our study found that DM was not related with AAA detection, consistent with a previous result on a negative association between DM and AAA growth.^24)^ Although smoking habituation and DM are well-known risk factors for atherosclerotic disease, the fact that only the former was a risk factor for AAA was interesting. At present, the implicated etiology of AAA is proteolysis, which is different from the pathophysiology of atherosclerosis. However, because aging, sex, and smoking habituation are risk factors for both atherosclerosis and AAA, atherosclerotic disease may overlap with AAA.

## Limitations

In this study, there might have been bias in the cohort selection. The study population cannot be considered healthy because all patients had reasons for undergoing ultrasound; therefore, the AAA prevalence in this study might be higher, compared with that in a healthy population. In addition, the use of direct interviews and medical records rather than diagnostic criteria to obtain information on comorbidities was a limitation. Given the large number of patients, it was possible that not all tests were performed by experienced laboratory technicians.

## Conclusions

Opportunistic AAA screening by ultrasound, including echocardiography, was efficacious and sufficient for the diagnosis of AAA. The prevalence of AAA was high in men and increased with age in both men and women. The risk factors that were strongly related with AAA were age, male sex, CAD, PAD, and smoking habituation.

## Availability Data and Materials

Deidentified patient data will be shared upon reasonable request. Please contact the corresponding author directly to request data sharing. Data will be available after publication until the end of December 2024. Access criteria for the data are limited to researchers who study in the same field. The data will be shared in Excel format via e-mail.

## Acknowledgments

We thank the staff of the 16 participating institutions, as follows: Ikuko Tashima, Junko Yonenuma, Ruriko Mikami, Keiko Itou, Yoko Oyama, Takako Osaka, Toru Murata, Kazunori, Akasaka, Toshiko Sasaki, Junko Sugai, Miki Saito, Tomoyuki Kazama, Maki Haga, Akiko Suga, and Chikako Shiine. We also sincerely thank Tomoe Tsukamoto for being the liaison among the institutions.

## Disclosure Statement

The authors declare no conflict of interest.

## Author Contributions

Study conception: FY

Data collection: FY

Analysis: GH

Investigation: FY and GH

Manuscript preparation: GH and FY

Funding acquisition: none

Critical review and revision: all authors

Final approval of the article: all authors

Accountability for all aspects of the work: all authors.
